# The Role of SARS-CoV-2 Testing on Hospitalizations in California

**DOI:** 10.3390/life11121336

**Published:** 2021-12-03

**Authors:** José Cricelio Montesinos-López, Maria L. Daza-Torres, Yury E. García, Luis A. Barboza, Fabio Sanchez, Alec J. Schmidt, Brad H. Pollock, Miriam Nuño

**Affiliations:** 1 Department of Public Health Sciences, University of California Davis, Davis, CA 95616, USA; mdazatorres@ucdavis.edu (M.L.D.-T.); ygarciapuerta@ucdavis.edu (Y.E.G.); aljschmidt@ucdavis.edu (A.J.S.); bpollock@ucdavis.edu (B.H.P.); 2 Centro de Investigación en Matemática Pura y Aplicada, Universidad de Costa Rica, San José 11502, Costa Rica; 3 Centro de Investigación en Matemática Pura y Aplicada—Escuela de Matemática, Universidad de Costa Rica, San José 11502, Costa Rica; luisalberto.barboza@ucr.ac.cr (L.A.B.); fabio.sanchez@ucr.ac.cr (F.S.)

**Keywords:** COVID-19, test positivity rate, mixed-effects model

## Abstract

The rapid spread of the new SARS-CoV-2 virus triggered a global health crisis, disproportionately impacting people with pre-existing health conditions and particular demographic and socioeconomic characteristics. One of the main concerns of governments has been to avoid health systems becoming overwhelmed. For this reason, they have implemented a series of non-pharmaceutical measures to control the spread of the virus, with mass tests being one of the most effective controls. To date, public health officials continue to promote some of these measures, mainly due to delays in mass vaccination and the emergence of new virus strains. In this research, we studied the association between COVID-19 positivity rate and hospitalization rates at the county level in California using a mixed linear model. The analysis was performed in the three waves of confirmed COVID-19 cases registered in the state to September 2021. Our findings suggest that test positivity rate is consistently associated with hospitalization rates at the county level for all study waves. Demographic factors that seem to be related to higher hospitalization rates changed over time, as the profile of the pandemic impacted different fractions of the population in counties across California.

## 1. Introduction

The SARS-CoV-2 virus, responsible for the novel coronavirus disease (COVID-19), was identified in late December 2019 in Wuhan, China [1], and spread rapidly, causing a global health crisis. As of 5 October 2021, more than 235 million cases and 4,812,221 deaths have been confirmed worldwide [2]. As the pandemic spread across the globe, governments started to enforce public policies to suppress SARS-CoV-2 transmission, including social distancing, contact tracing, stay-at-home orders, school closings, limited public space utilization, and border closures [3,4]. To date, public health officials continue to promote some of these non-pharmaceutical measures, mainly due to delays in mass vaccination and the growing number of new COVID-19 variants [5]. Mass surveillance testing, efforts of isolation, quarantine, and contact tracing became essential control measures for curtailing the burden of the COVID-19 pandemic [6]. The successful epidemic control measures taken by countries such as Korea, Taiwan, Japan, China, New Zealand, and the Czech Republic, which emphasized high testing rates during the initial stages of the pandemic, supported the proposal that mass surveillance testing could help limit viral transmission when properly leveraged [7,8,9,10,11]. However, it remains unknown which testing strategies are the best and whether different approaches show significant and measurable effects on viral spread in general and the rates of severe or deadly cases in particular [12]. Although population-scale testing is proven to reduce SARS-CoV-2 [13], it appears to become less effective as viral prevalence decreases and is insufficient to eliminate viral transmission on its own [14,15].

Public health officials commonly use the test positivity rate to infer the adequacy of population-level testing and the rate of COVID-19 transmission in a population [16]. A low test positivity rate indicates low viral prevalence and a testing program with sufficient surveillance capacity. In contrast, a high test positivity rate suggests that the amount of testing is insufficient and that many infected people go unnoticed, especially when test positivity rates are higher than the expected prevalence [17]. Implementing mass testing may also lead to fewer hospitalizations by reducing new infections by offering interventions for symptomatic and asymptomatic cases discovered early [6,18,19]. Hospitalization is also influenced by the demographic structure of the population and health care system factors. In theory, a public health system that is better prepared to identify and support the isolation of cases discovered by surveillance testing and treat those who require medical care should result in lower hospitalizations rates.

On 26 January 2020, the first documented case of COVID-19 in California occurred in Orange County [20]. Since then, the state government has implemented a variety of strategies to contain the spread of the virus [21]. On 4 March 2020, California declared a state of emergency, followed by a mandatory statewide stay-at-home order on 19 March 2020. On 18 June 2021, a statewide mask mandate was ordered due to the rising number of cases and deaths. These mandates were in force until 15 June 2021, when California started reopening the economy [22], with 70% of those eligible having at least one dose of the COVID-19 vaccine and more than 40% of the population fully vaccinated [23]. As of 22 September 2021, California has had three COVID-19 case waves. The first peak occurred in mid-July 2020, reaching an average of 10,000 new cases per day (first wave, May–September 2020) [24]. During this first wave, most infections were geographically concentrated in the Central Valley, primarily dominated by agriculture, manufacturing services, and retail, meaning few residents could make the transition to working from home [25]. In Autumn 2020, COVID-19 cases spiked again, to a peak of 40,000 new cases per day at the end of December (second wave November–January 2021). During this wave, Los Angeles was one of the main epicenters of the pandemic [26,27]. The third wave associated with the SARS-CoV-2 delta variant started in mid-June 2021 after the lifting of the statewide stay-at-home order. By mid-September, the number of reported daily COVID-19 infections was decreasing, and, as of 20 September 2021, California reported the lowest coronavirus state incidences case rate in the U.S. [28,29]

In this paper, we aim to provide an exploratory data analysis to verify how demographics and positivity rate correlate with COVID-19 hospital admission in California. The analysis was performed in each of the three waves, using a mixed linear model and data related to hospitalizations for COVID-19, age, race, ethnicity, poverty, and mobility.

## 2. Materials and Methods

The main goal of this analysis is to describe the effect of surveillance testing on hospitalizations for COVID-19. We performed a comparative analysis using a mixed linear model to study the relationship between hospitalization rates for COVID-19 and positive cases, diagnostic tests, mobility, age, race and ethnic group, poverty, and education across the counties of California. Sixteen of the fifty-eight counties were excluded from this analysis: Calaveras, Colusa, Del Norte, Glenn, Inyo, Lassen, Mariposa, Modoc, Mono, Plumas, San Benito, and Siskiyou, due to low quality of hospital and mobility data; and rural counties like Alpine, Sierra, Sutter, and Trinity because they do not have hospital wards, so patients from those counties would go to neighboring counties for COVID-19 medical care.

**Figure 1:**
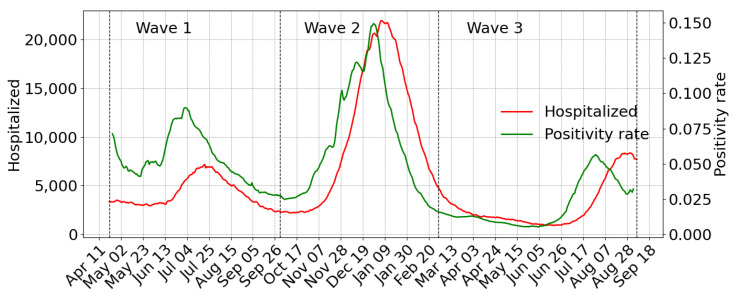
Positivity rate (7-day moving average) and the number of patients hospitalized in an inpatient bed who have laboratory-confirmed COVID-19 in California.

We analyzed three waves according to the three primary outbreaks reported in California [30]. We defined the first wave period from 21 April 2020 to 30 September 2020; the second wave starts on October 1 and ends on 28 February 2021, and the third starts on March 1 and ends on September five, 2021 (Figure 1).

### 2.1. Data Sources

**Figure A1:**
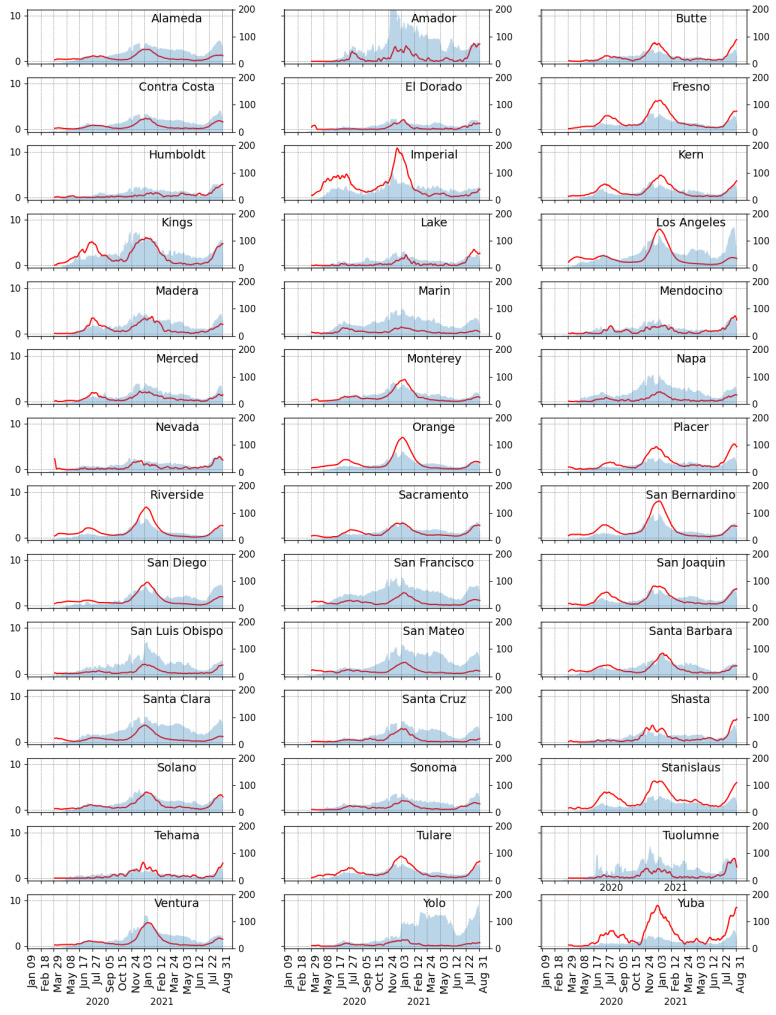
This figure displays the number of patients hospitalized in an inpatient bed who have laboratory-confirmed COVID of the California counties [55] considered in the study and testing per 10 K population from 5 January 2020 to 6 September 2021.

**Figure A2:**
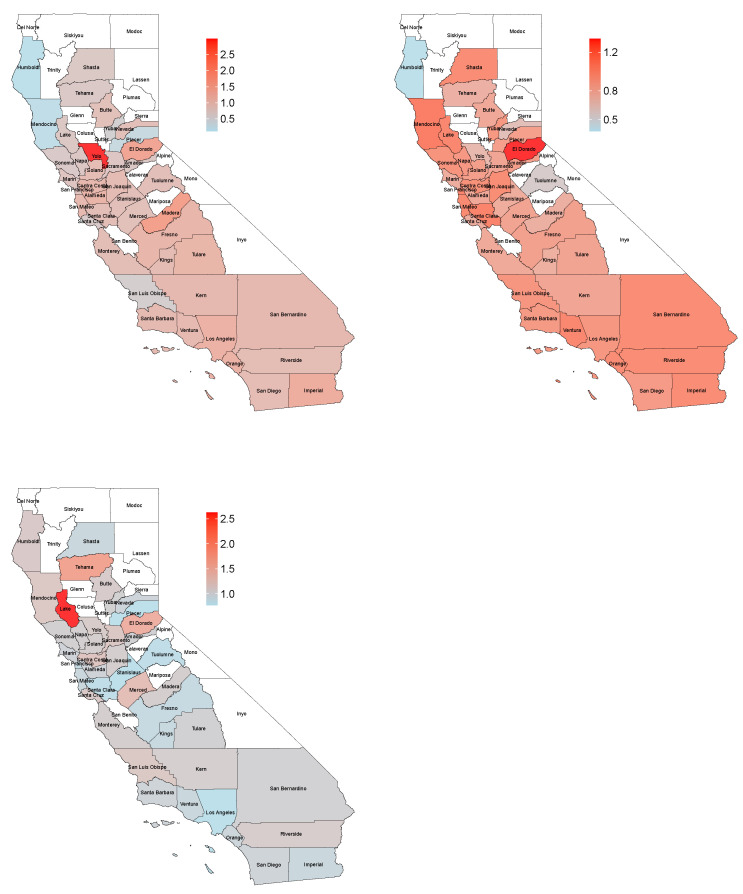
Values in the county-level maps represent the effect of the positivity rate on hospitalizations at each county for each wave. **Top Left**: Wave 1. **Top Right**: Wave 2. **Bottom**: Wave 3.

We used publicly available epidemiological data for COVID-19 daily cases and hospitalization admissions at the county level from the official website of the California Department of Public Health (CDPH) [31]. We refer to confirmed cases as the total number of laboratory-confirmed COVID-19 cases at the specified episode date. Episode date, when available, corresponds to the earliest of the following dates: date received, date of diagnosis, date of symptom onset, specimen collection date, or date of death. A hospital admission corresponds to the event when a patient is admitted in the inpatient setting at a hospital or ICU (including medical surgical units) and has a laboratory-confirmed COVID-19 diagnosis. In-hospital admissions do not include patients in affiliated clinics, outpatient departments, emergency departments, and overflow locations awaiting an inpatient bed. Data from the American Community Survey (ACS) [32] estimates characteristics at the county level for age and race or ethnic group. We used the Healthy Places Index (HPI) to account for community-level factors contributing to social vulnerability. The HPI is produced by the Public Health Alliance of Southern California, which combines twenty-five community characteristics (e.g., the number of people living below the poverty line, the number of people with lower levels of education, areas with more renters and fewer homeowners, among others) into a single index value to account for the level of poverty, education, and life expectancy in a particular community [33]. The degree of intra-community mobility was produced from Google’s Community Mobility Reports [34]. Six Google-specific data streams (grocery and pharmacy, parks, residential, retail and recreation, transit stations, and workplaces) were combined to obtain a single mobility measure for the county using principal component analysis (see Appendix A.2 for details). All data that changed over time were analyzed weekly to minimize fluctuations observed at the daily level. We considered 7-day averages for daily test positivity rate, intra-community mobility, and hospitalization rate (see Figure A1 and Figure A2), given that this is likely to be less volatile.

### 2.2. Exposure and Outcome

**Figure 2:**
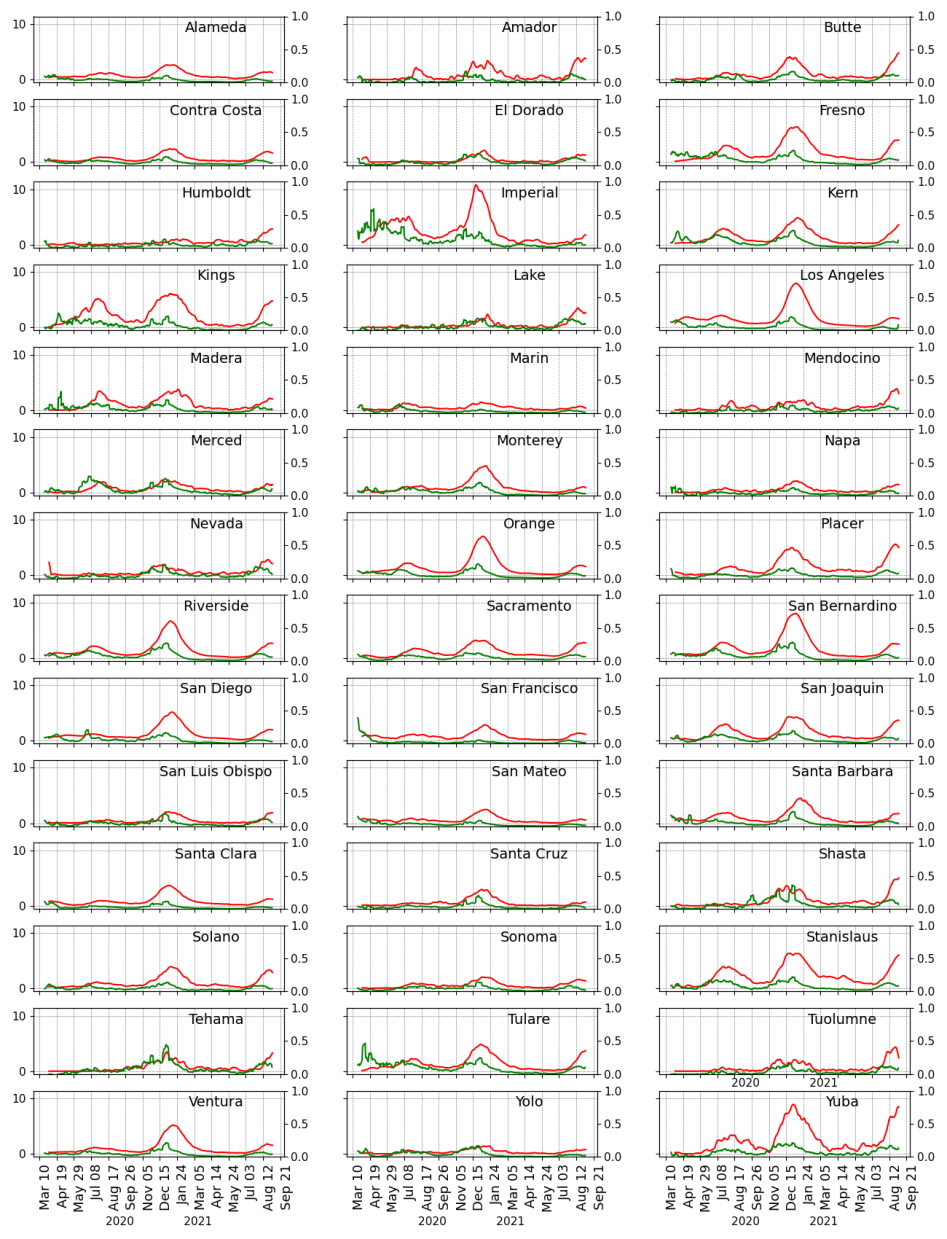
Hospitalization rate per 10,000 county residents (red line) and positivity rate (7-day moving average, green line) from 28 March 2020 to 6 September 2021.

The number of tests completed and the number of positive cases captured is not meaningful without further specification. The number of confirmed cases on a given day is related to the actual prevalence, the average duration of disease, and the gross number of tests performed, such that an increase in the number of tests can reveal more existing infections and a change in estimates of the prevalence. Test positivity rate incorporates both the number of tests done and the number of positive cases discovered, frequently used for monitoring the progression of the COVID-19 pandemic [35,36], and its correlation with hospitalization rates has been shown in previous studies [37,38] consistent with our use here. We calculated the average positivity rate at the county level by dividing the 7-day average of daily confirmed cases by the 7-day average of daily tests. The hospitalization rate was conceptualized as the average weekly hospital admission rate for laboratory-confirmed COVID-19 per 10,000 county residents, see Figure 2. The weekly average positivity and hospitalization rates were log-transformed to capture the effect of detected infections and testing on COVID-19 hospitalizations. It is expected that a patient that is hospitalized will likely be admitted several days after a confirmed COVID-19 diagnosis. This implies that the number of hospitalizations reported on a specific day will be delayed. This study assumed a two-week delay between symptom onset and hospitalization as it provided the best fit for the correlation at the county-level.

### 2.3. Model

Hospitalization data are made up of repeated measurements. The first, second, and third waves represent 24, 22, and 27 measurements of hospitalization rate, respectively, corresponding to the number of weeks in each wave. The traditional linear regression model is not appropriate for studying data with multiple repeated measures [39]. Therefore, we employed a linear mixed-effects model that incorporates repeated observations at the county level.

Let $Y_{j}$ be the I×1 dependent variable corresponding to the log of the rate of hospital admissions for COVID-19 per 10,000 inhabitants at the county *j*. The subscripts j=1,2,...,J and i=1,2,...,I represent the 42 counties in California and the number of weeks in the wave data collected, respectively. $X_{j}$ is the I×p fixed-effects design matrix; β is the p×1 fixed-effects vector; $Z_{j}$ corresponds to I×q matrix of random-effects design matrix; $u_{j}$ represents the q×1 vector of random effects and $\varepsilon_{j}$ is the I×1 vector of residuals. $u_{j}$ is independent of $\varepsilon_{j}$. **G** is the q×q covariance matrix for the random effects, and $R_{j}$ is the I×I covariance matrix for the residuals. The model we considered includes a random intercept and a random slope concerning the positivity rate (q=2) since we hypothesize that each county has a different baseline positivity rate and that the effect of the positivity rate on hospitalization differs between counties.

We define the general form of the mixed linear regression model as follows:

(1) $$\begin{array}{cc} Y_{j} & =X_{j}\beta +Z_{j}u_{j}+\varepsilon_{j}\\ u_{j} & ∼N\left(0,G\right)\\ \varepsilon_{j} & ∼N\left(0,R_{j}\right). \end{array}$$

The term $X_{j}\beta$ corresponds to the fixed effect(s) component (a standard general linear model) and $Z_{j}u_{j}$ to the random effects. The model was fitted using the lmer function in the lme4 package for R [40].

Since only the hospitalization rate and the positivity rate were log-transformed, we interpret the coefficient ($\beta_{r}$) for the log positivity rate as the percent increase in the hospitalization rate for every 1% increase in the positivity rate. The estimation for all other coefficients ($\beta_{p}$’s) requires transformation via $100\times (\exp\left(\beta_{p}\right)-1)$, which gives the percent increase (or decrease) in the hospitalizations rate for every one-unit increase in the independent variable.

### 2.4. Variable Correlation

**Figure 3:**
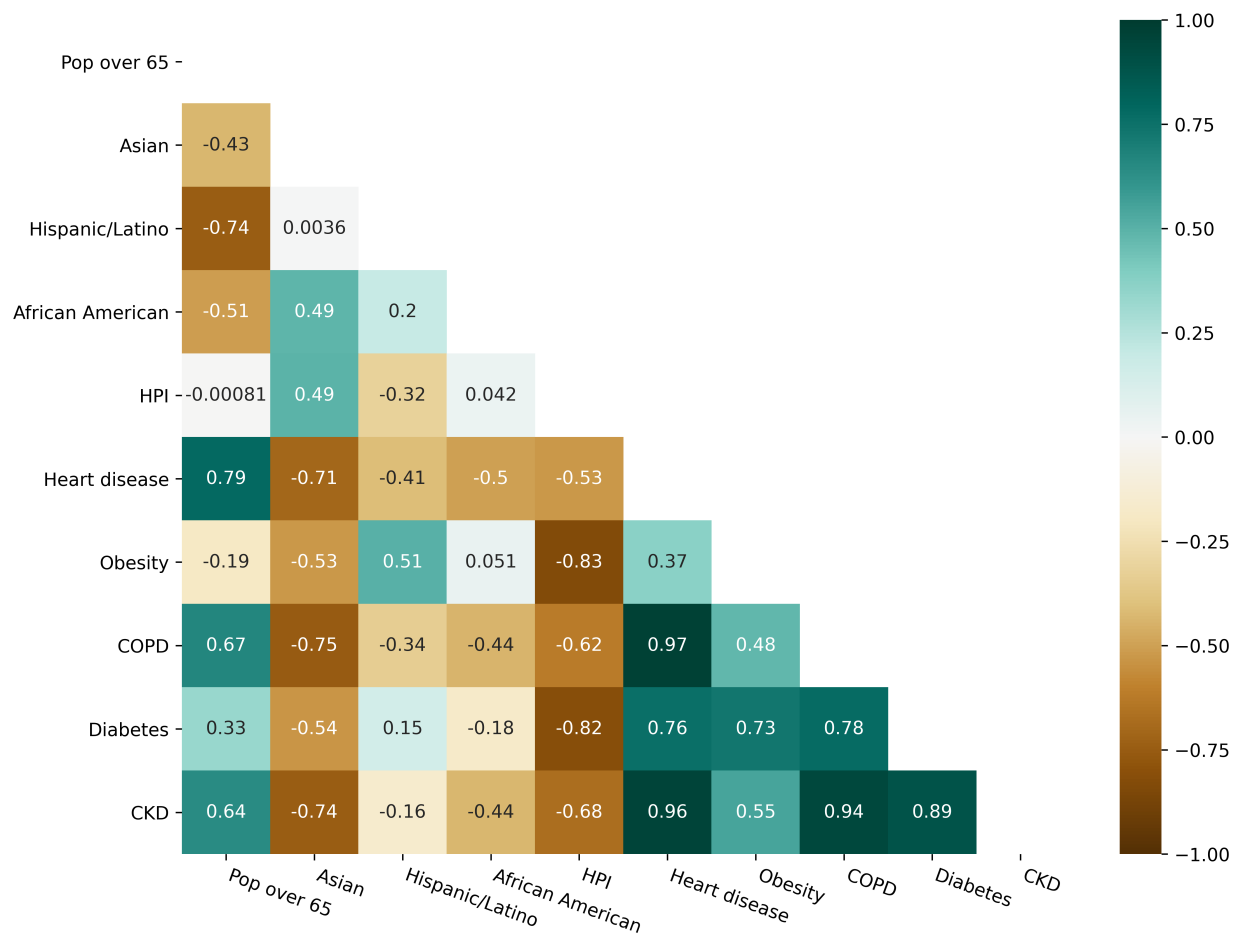
Correlation matrix of the demographics variables and comorbidities.

We considered several independent variables in building the model and explored multicollinearity (see variables description in Table A1 and Table A2) among them to determine variables to be included in the model. We calculated and plotted the Pearson correlation coefficient for the variables of interest. Figure 3 highlights the weaker correlation across demographic variables while finding high correlations of the comorbidities between them and most of the demographic variables.

We described the presence of multicollinearity using the variance inflation factor (VIF). Values of VIF that exceeded 10 were regarded as variables with multicollinearity. Table A3 describes very large values of the VIF and, after removing independent variables with significant VIF values, we are left with only the demographic variables given in the Table A1. Given the findings of the correlation analysis, we excluded disease prevalence variables from the final model.

## 3. Results

The coefficient estimates and the 95% confidence intervals (CI) for the linear mixed model are presented in Table 1. The β value represents the effect that each variable has on the hospitalizations rate. Variables with a *p*-value <0.05 were considered statistically significant. Results show that significant variables changed over time, but the positivity rate consistently remained significant across all three waves with a coefficient $\beta_{r}$ close to one. Regarding hospitalization rates for different racial and ethnic groups, counties with a higher population percentage of non-White race or ethnic groups had higher hospitalization rates in the first and second waves, see Table 1. In the first wave of infections, counties saw an average 7.4% increase in hospitalization rate for every 1% of the population identified as Hispanic or Latino, and a 16.6% increase in hospitalization rate for every 1% of the population that identifies as African American. In the second wave, counties with high proportions of Hispanic or Latino and African American populations were not significantly different, but a 3.4% increase in hospitalization rates was associated with every 1% of the population that identifies as Asian.

HPI was significant and positive in the first wave, meaning that counties with more significant economic, social, and healthcare resources reported increased hospitalization rates compared to counties with fewer resources. Higher intra-community mobility was associated with higher hospitalization rates; however, in the second wave, we found that higher mobility was negatively associated with hospitalization rates.

Table 2 displays the coefficient value related to the log positivity rate for each county in the three waves. These values are equal to ($\beta_{r}+u_{r_{j}}$), where $\beta_{r}$ correspond to the general coefficient for the log positivity rate (Table 1) and $u_{r_{j}}$ is the random coefficient for the j-th county, j=1,2,...,J. In Table 2, counties with higher coefficient values had stronger associations between test positivity rate and hospitalization rate.

## 4. Discussion

A mixed linear model was used between the COVID-19 hospitalization rate and factors such as age, ethnicity, race, poverty index, and intra-community mobility. Our primary interest was studying the impact of testing rates on county-level hospitalization rates, as county health departments were usually responsible for public testing administration. We found that the test positivity rate was consistently significant and positively associated with the hospitalization rate during all three waves of COVID-19. Hospitalization rate increased at an almost 1:1 basis with a positivity rate. While other possible predictors of hospitalization rate, including the density of different race or ethnic groups, social vulnerability, and intra-community mobility, had pronounced effects at differing times during the pandemic, none were consistent predictors of hospitalization rate for all three waves of infection.

The actual local prevalence and the number of tests administered both affect the positivity rate value. Generally, the higher the true prevalence, the higher the positivity rate will be; as more tests are deployed, the positivity rate will converge with the true prevalence. The nature of diagnostic testing on a first-come-first-served basis frequently leads to positivity rates more than the actual prevalence if testing rates are insufficient to sample the mild or asymptomatic cases. In other words, if the number of tests is a limiting factor, and they are used primarily to confirm likely cases more frequently than a random surveillance sampling of the population, positivity rates will be biased upwards compared to the actual prevalence. This assumes that those who suspect they have the disease or suspect exposure are more likely to seek a test than those who have no such suspicion. Thus, high test positivity rates are likely a mix of biased sampling and high prevalence, but clarifying which is dominant during a specific time frame requires high-quality auxiliary data that may not exist. Our results suggest that actions that reduce the test positivity rate are likely to reduce the hospitalization rate by a similar magnitude. Simply increasing the number of tests will only significantly reduce the positivity rate if sampling bias is the dominant reason for a high positivity rate. Determining the effect on hospitalization rate of reducing test positivity rate in bias-dominant versus prevalence-dominant systems is beyond the scope of this paper, but remains an important question.

The response following detection is essential. Theoretically, early detection of a new case, symptomatic or asymptomatic, and rapid isolation will prevent further potential hospitalizations. Extrapolating from our results, we expect that the counties that more regularly tested a more significant proportion of their population—from asymptomatic surveillance or robust testing requirements for essential workers—experienced lower hospitalization rates than the counterfactual scenario. However, care must be taken extending this reasoning too far: large-scale population testing can theoretically lead to reduced hospitalizations, but the effect will always be indirect. The resources and infrastructure must support proper mass testing and preparation to respond to the information garnered from the testing program, which no two counties will have done identically, hence why each county reported here maintained intercepts that varied from each other over time.

A low positivity rate due to a high amount of testing does not always imply adequate pandemic control. Not only does the gap between testing rates among suspected cases, known exposures, and the unexposed or asymptomatic matter, but testing rates among different demographic groups demonstrably effect the value of testing data. Suppose the mass testing systemically excludes people with a high-risk profile (as could quickly occur where healthcare accessibility is low). In that case, many infections could remain undiscovered for long periods, leading to a growth in the hospitalization rate despite low positivity rates. The pandemic has not affected everyone equally. Disparities in coronavirus disease outcomes by racial and ethnicity as well as socioeconomic status have been reported since the beginning of the pandemic [41]. Our findings highlight that areas with larger relative populations of Hispanic or Latinos and African Americans were significantly correlated with higher hospitalization rates in the first wave and with Asians in the second wave, consistent with previous studies [42,43,44]. The underlying causes of health disparities in Latinos, African Americans, and poor communities are related to social and structural determinants of health [45]. Implementing social distancing, especially at the beginning of the pandemic, may have been challenging because these communities, on average, live in more crowded conditions and work more frequently in essential public-facing occupations. In addition, their access to health services is systemically limited, so that populations have a disproportionate burden of underlying comorbidities and lack the possibility of accessing adequate and timely treatment when affected by the SARS-CoV-2 virus [46], and possibly confounding the relationship between test positivity rates and hospitalization rates, as discussed above.

The HPI is correlated positively with the hospitalization rate in the first wave, which implies that counties with higher socioeconomic status had a higher probability of reporting hospitalizations. One of the reasons may be the capacity and better availability of hospital facilities attributed to economic resources. Mobility was another significant variable that positively and negatively correlated with the hospitalization rate in the first and second waves. A similar result was reported in [47] for COVID-19 transmission and mortality rates. Early in the pandemic, mobility patterns were drastically affected by containment measures implemented to slow the spread of the disease. Our results show a linear correlation between mobility and the rate of hospitalization in the first wave, in agreement with previous reports [48], which implies that an increase in the circulation of people could cause an increase in infections and, consequently, in hospitalizations. However, it is not clear how mobility affected the growth rates of the COVID-19 infection once the lockdowns were lifted because other interventions became more widely available and easier to adhere to, such as wearing face masks and social distancing, patterns of both mobility and growth of infections became non-linear [48]. One interpretation could be that areas with lower infection rates allowed for greater freedom in summer activities, negatively correlating positivity and hospitalization rates. Care must be taken in attributing causation to relationships between these covariates and hospitalization rates without further study.

This study has some limitations that are important to consider. First, it is focused on county-level analysis and is intended to investigate population-level risk; conclusions at the individual level are not appropriate and should not be applied. Second, as discussed earlier, we did not attempt to address whether a given data point on test positivity was produced during a bias-dominant or prevalence-dominant period. Thus an unknown proportion of the relationship between positivity rate and hospitalization rate is likely due to natural increases in the prevalence. Third, the hospitalization rate is also dependent on available hospital beds, which we did not consider as a factor given the limited availability and reliability of such data at the county level. Thus, some instances where hospitalization would have been an outcome for a patient except for bed availability were not accounted for, which could have led to point underestimates of our primary outcome measurements.

Knowing the factors that affect the spread of the virus and hospitalizations allows local decision-makers to help identify areas at higher risk for severe COVID-19 and guide resource allocation and implementation of prevention and mitigation strategies. These findings highlight how the most significant factors impacting hospitalizations have changed with the pandemic’s evolution. The positivity rate is the only factor to prevail over time as a significant and directly correlated with hospitalization rate.

## Figures and Tables

**Table 1:** Association between hospitalization rates and independent variables at the county level.

	1st Wave		2nd Wave		3rd Wave	
**Variable**	Estimate * (95% CI)	***p*** **-Value**	Estimate * (95% CI)	***p*** **-Value**	Estimate * (95% CI)	***p*** **-Value**
Positivity rate	0.9 (0.6, 1.4)	<0.001	0.9 (0.8, 0.9)	<0.001	1.1 (0.9, 1.2)	<0.001
Pop over 65	−13.2 (−30.6, 8.5)	0.246	3.1 (−3.8, 10.6)	0.416	−7.7 (−15.1, −0.1)	0.060
Asian	0.8 (−7.2, 9.4)	0.861	3.4 (0.7, 6.1)	0.020	−0.5 (−3.9, 2.9)	0.763
Hispanic/Latino	7.4 (2.1, 12.9)	0.010	0.9 (−0.6, 2.5)	0.259	−0.6 (−2.3, 1.3)	0.535
African American	16.6 (0.1, 35.9)	0.070	0.1 (−4.7, 5.1)	0.982	1.8 (−4.3, 8.2)	0.582
HPI **	4.9 (2.4, 7.4)	<0.001	−0.4 (−1.2, 0.3)	0.266	0.2 (−0.7, 1.2)	0.686
Mobility	4.9 (1.9, 7.9)	<0.001	−2.8 (−3.4, −2.1)	<0.001	0.1 (−1.7, 1.8)	0.950

* A 1% increase in the positivity rate coefficient consistently corresponds to around a 1% percent increase in the hospitalization rate. Interpretation for the other independent variables is a one-unit increment corresponding to a percent change, namely positive or negative, depending on the coefficient sign. ** HPI: Healthy Places Index.

**Table 2:** Estimates of the association between test positivity rate and hospitalization rate for each wave of study.

County	Wave 1	Wave 2	Wave 3	County	Wave 1	Wave 2	Wave 3
Yolo	2.99	0.70	1.10	Santa Clara	0.95	1.08	0.82
Madera	1.64	0.77	1.05	Stanislaus	0.92	0.80	0.78
El Dorado	1.52	1.34	1.53	Nevada	0.92	0.64	0.94
Imperial	1.34	1.02	0.92	Tuolumne	0.91	0.55	0.80
Los Angeles	1.23	0.95	0.78	Butte	0.90	0.82	1.10
Orange	1.22	1.02	0.94	Merced	0.90	0.86	1.30
Alameda	1.18	0.82	0.98	San Francisco	0.82	1.06	0.87
Tulare	1.15	0.86	1.00	Marin	0.79	0.79	0.98
Fresno	1.14	0.88	0.87	Sacramento	0.75	0.85	1.16
Contra Costa	1.09	0.99	1.21	Napa	0.73	0.95	1.04
San Bernardino	1.06	1.01	1.02	Lake	0.71	1.06	2.62
Kern	1.06	0.84	1.05	Amador	0.71	0.90	1.01
San Mateo	1.04	1.03	0.91	Shasta	0.70	1.02	0.91
Ventura	1.04	1.05	0.91	Tehama	0.69	0.76	1.70
Santa Barbara	1.04	0.93	0.97	Sonoma	0.67	0.97	1.03
San Joaquin	1.01	1.01	1.06	Santa Cruz	0.66	0.79	1.12
Kings	1.00	0.77	0.89	San Luis Obispo	0.55	0.95	1.15
San Diego	0.99	0.92	0.97	Yuba	0.52	0.95	1.02
Solano	0.99	0.90	1.06	Placer	0.29	0.89	0.77
Riverside	0.97	1.02	1.09	Mendocino	0.18	1.10	1.17
Monterey	0.95	0.81	1.05	Humboldt	0.12	0.38	1.14

## Data Availability

The datasets used can be found at https://github.com/Cricelio23/The-Role-of-SARS-CoV-2-Testing-on-Hospitalizations-in-California (accessed on 5 October 2021).

## Appendix A. Variable Description

We describe the co-variable used in the analysis; mobility, age, race, ethnicity, poverty, and education in the California counties.

### Appendix A.1. Demographic Variables

Demographic characteristics such as age, ethnicity, and race by county was obtained from the United State Census Bureau [32].

**Table A1:** Demographic variables considered in the mixed linear model.

Variable	Description
Pop over 65	Percentage of population ages 65 and above
Asian	Percentage of Asian population
Hispanic/Latino	Percentage of Hispanic or Latino population
African American	Percentage of Black or African American population
HPI	Healthy Places Index

**Table A2:** Description of comorbidity variables.

Variable	Description
Heart disease	Prevalence of heart disease
Obesity	Prevalence of obesity
COPD	Prevalence of chronic obstructive pulmonary disease
Diabetes	Prevalence of diabetes
CKD	Prevalence of chronic kidney disease

**Table A3:** Variance inflation factor (VIF).

Variable	VIF
Pop over 65	299.79
Asian	11.19
Hispanic/Latino	73.89
African American	6.50
HPI	11.71
Heart disease	3846.96
Obesity	235.79
COPD	1648.60
Diabetes	1430.87
CKD	3342.10

### Appendix A.2. Mobility Trends

The existence of social contacts could be proxied by mobility data [49,50,51,52], with frameworks such as Google’s Community Mobility Report [34], COVID-19—Mobility Trends Reports—Apple [53] and Safegraph [54] being able to measure mobility, as it measures citizens’ mobility according to different types.

Our analysis is based on 40 counties of California for which both hospitalization and Google mobility data were available. Google mobility data included six data-streams: grocery and pharmacy, parks, residential, retail and recreation, transit stations, and workplaces. We combined all Google-specific data streams to obtain a google county mobility measure. We used an unsupervised machine learning method known as principal component analysis to construct the google mobility index using the six mobility metrics. The first principal component explained more than 50% of the variability in the data by each county, indicating a good dimension reduction (Table A4).

A regression analysis was used to estimate the lag length. The results show that mobility is correlated with COVID-19 hospitalizations in most counties with lags of 3–4 weeks.

**Table A4:** The variance explained by the first principal component for the Google’s Community Mobility Report.

County	Explained Variance	County	Explained Variance	County	Explained Variance
Alameda	0.62	Mendocino	0.60	San Mateo	0.65
Amador	0.43	Merced	0.68	Santa Barbara	0.67
Butte	0.56	Monterey	0.66	Santa Clara	0.60
Contra Costa	0.63	Napa	0.56	Santa Cruz	0.60
El Dorado	0.50	Nevada	0.58	Shasta	0.51
Fresno	0.67	Orange	0.68	Solano	0.60
Humboldt	0.60	Placer	0.50	Sonoma	0.66
Imperial	0.67	Riverside	0.65	Stanislaus	0.62
Kern	0.60	Sacramento	0.66	Tehama	0.4
Kings	0.61	San Bernardino	0.62	Tulare	0.57
Lake	0.65	San Diego	0.71	Tuolumne	0.47
Los Angeles	0.73	San Francisco	0.73	Ventura	0.56
Madera	0.51	San Joaquin	0.68	Yolo	0.66
Marin	0.62	San Luis Obispo	0.65	Yuba	0.54

## Appendix C. Maps

This maps displays the coefficient value related to the log positivity rate for each county in the three waves.
